# Methylation Mediated Silencing of miR-155 Suppresses the Development of Preeclampsia *In Vitro* and *In Vivo* by Targeting FOXO3

**DOI:** 10.1155/2022/4250621

**Published:** 2022-05-26

**Authors:** Xiaohua Luo, Ci Pan, Xiaopei Guo, Cunhua Gu, Yulian Huang, Jing Guo, Ying Zeng, Jinjing Yue, Shihong Cui

**Affiliations:** ^1^Department of Obstetrics and Gynaecology, The Third Affiliated Hospital of Zhengzhou University, Zhengzhou, 450052 Henan, China; ^2^Henan Provincial Clinical Research Center for Perinatal Medicine, The Third Affiliated Hospital of Zhengzhou University, Zhengzhou, 450052 Henan, China

## Abstract

Preeclampsia (PE) is a common pregnancy-related syndrome characterized by chronic immune activation. This study is aimed at exploring the role of miR-155 in the inflammatory pathogenesis of PE. Placental tissues and peripheral blood were collected from all subjects. BSP detection analysis was performed to evaluate miR-155 methylation levels. ELISA was performed to measure the levels of inflammatory cytokines and MMP2 in serum samples and cellular supernatants. HTR-8/SVneo and JEG-3 cells were transfected with miR-155 mimic and the inhibitor to establish the overexpressed miR-155 and silenced miR-155 cell models, respectively. Treatment with 5-Aza was performed to alter the DNA methylation level of miR-155. The PE rat model was established after subcutaneous injection of NG-nitro-L-arginine methyl ester. The CCK-8 assay, TUNEL staining, and Transwell assay were performed. Reverse transcription-quantitative PCR, Western blot analysis, and immunohistochemical assay were used to analyze related gene expression levels. The luciferase reporter assay was used to investigate the direct interaction between FOXO3 and miR-155. Results showed that miR-155 was remarkably upregulated and inversely correlated with the promoter methylation level in the placental tissue from PE patients. The *in vitro* experiments indicated that miR-155 decreased viability, migration, and invasion, but increased apoptosis in trophoblast cells. FOXO3 was confirmed as the target of miR-155. Transfection of the miR-155 inhibitor suppressed inflammation and oxidative stress, but elevated proliferation, migration, and invasion of trophoblast cells, which were abolished by 5-Aza treatment or cotransfection with si-FOXO3. In summary, our data suggested that methylation-mediated silencing of miR-155 can inhibit the apoptosis, inflammation, and oxidative stress of trophoblast cells by upregulating FOXO3.

## 1. Introduction

Preeclampsia (PE) is one of the most frequently occurring pathological complications of pregnancy with a worldwide prevalence of 2-8% [1]. Diversified factors, including impaired oxygen dysregulation, placental oxidative stress, chronic immune activation, and spiral artery remodeling, are involved in the pathophysiological characteristics of PE [2–4]. During normal pregnancy, acquisition of invasive capabilities by trophoblasts plays an important role in spiral artery remodeling and sufficient blood perfusion [5]. That is, the proliferation, migration, and invasion of trophoblast cells into the uterus contribute to human placental formation and embryo implantation [6]. Therefore, investigating the underlying molecular mechanisms associated with impaired trophoblastic proliferation and invasion will help to provide more effective treatments for PE.

MicroRNAs (miRNAs) are a class of small noncoding RNAs (21-23 nucleotides) that negatively regulate gene expression by directly binding with the 3′-untranslated region (3′-UTR) of the target mRNAs [7]. miRNAs are frequently aberrantly expressed in placental tissues and participate in different aspects of placentation, including trophoblast proliferation, apoptosis, differentiation, invasion, inflammatory response, and oxidative stress [8–12]. miR-155 as the product of the polygenic conserved region of the B-cell integration cluster gene is composed of 3 exons and is located in chromosome 21q21 [13]. Evidence indicates that miR-155 is upregulated in the placentas in a large number of pregnant women suffering from PE [14]. Yang et al. [15] also showed that the level of miR-15 expression was increased and positively correlated with IL-17 levels in PE placentas and serum, compared with the normal group. Despite extensive knowledge of miR-155 dysregulation, little is known about the mechanism that controls expression of miR-155 in PE. One possibility is that DNA methylation may be involved in this process, which occurs at the sites of CpG dinucleotides and plays a critical role in the regulation of gene expression [16].

Functionally, overexpression of miR-155 contributes to PE development by targeting and downregulating the angiogenic regulating factor CYR61 [13]. The study by Li et al. [17] revealed that miR-155 has a negative regulatory role in the migratory behavior of HTR-8/SVneo cells via modulating eNOS. Similarly, Dai et al. [18] demonstrated that miR-155 inhibits the proliferation and invasion of HTR-8/SVneo cells by downregulating cyclin D1. These data collectively demonstrate that miR-155 decreases cell proliferation, migration, and invasive trophoblast cells. However, knowledge about the role of miR-155 in the inflammatory pathogenesis of PE remains largely unclear. The Forkhead-box class O transcription factor 3 (FOXO3), an essential member of the FOXO family, regulates the proliferation of immune cells such as B lymphocytes and T lymphocytes [19]. Previous studies showed that the miR-155/FOXO3 axis is closely related to the occurrence and development of different diseases. For instance, miR-155 contributes to drug resistance by targeting FOXO3 expression in colon cancer [20] and pancreatic cancer [21]. Importantly, miR-155 appears to play a role in the intestinal inflammation of patients with active ulcerative colitis by downregulating expression of FOXO3 [22]. miR-155-5p promotes fibroblast cell proliferation by negative modulation of FOXO3 in vulvar lichen sclerosis, a chronic inflammatory skin disorder [23]. A recent study by Zhou et al. [24] further demonstrated that inhibition of the miR-155/FOXO3 axis is involved in the tanshinone IIA ameliorating inflammation response in osteoarthritis patients. Considering that the inflammatory response plays a critical role in the pathogenesis of PE, the present study aimed to examine whether FOXO3 was the direct downstream regulator of miR-155 during the inflammatory pathogenesis of PE.

Here, we first used BSP detection analysis to quantify the methylation levels of upstream regions of miR-155 in PE patients to determine whether they are methylated in this disorder. Next, we investigated the functional role of miR-155 in trophoblast cells by RNA interference or altering the methylation status. Moreover, we evaluated the miR-155/FOXO3 signaling in the inflammation pathogenesis of PE *in vitro* and *in vivo*.

## 2. Materials and Methods

### 2.1. Clinical Samples

Forty pregnant women (20 patients diagnosed as PE and 20 age-matched non-PE) hospitalized in The Third Affiliated Hospital of Zhengzhou University between August 2017 and November 2019 were enrolled in this study after signing a written informed consent. The diagnosis of PE was based on diagnosis and treatment of hypertension and preeclampsia in pregnancy: a clinical practice guideline in China (2020) [25]. When the placenta was delivered, tissues were immediately collected from the mother's surface of the placent, which were then snap-frozen in liquid nitrogen. Also, peripheral blood (5 mL) was collected in vacuum vasculature (Qiagen GmbH) from all patients. Serum was acquired by centrifugation at 3,000 × g for 10 min at 37°C. The study protocol was approved by the Ethics Committee of The Third Affiliated Hospital of Zhengzhou University.

### 2.2. Cell Culture

Human trophoblast cell lines (HTR-8/SVneo and JEG-3) and 293 T cells were purchased from the Cell Bank of The Chinese Academy of Sciences. All cell lines were cultured in RPMI 1640 medium (Gibco, Shanghai, China) supplemented with 10% FBS and 1% penicillin/streptomycin in an incubator with 5% CO_2_ at 37°C.

### 2.3. Oligonucleotide Transfection

The miR-155 mimic, the miR-155 inhibitor, negative control (NC), si-NC, and si-FOXO3 were synthesized by Shanghai GenePharma, Ltd. (Shanghai, China). Then transfection was performed in HTR-8/SVneo and JEG-3 cells with the above oligonucleotide for 48 h using Lipofectamine 2000 (Invitrogen, Carlsbad, CA) according to the manufacturer's protocol. In the rescue experiments, HTR-8/SVneo and JEG-3 cells were transfected with si-FOXO3, followed by transfection with the miR-155 inhibitor. Subsequently, cells were harvested for further analysis.

### 2.4. Treatment with 5-Aza

To block DNA methylation, HTR-8/SVneo and JEG-3 cells were treated with 2.5 *μ*mol/l 5-aza-2′-deoxycytidine (5-Aza; Sigma-Aldrich, MO, USA) for 3 days. Then, cells were transfected with the miR-155 inhibitor as mentioned above, during which time the culture medium was replaced every 24 h.

### 2.5. Cell Viability Assay

The cell viability of human trophoblast cells was evaluated using a Cell Counting Kit-8 (CCK-8; Dojindo Molecular Technologies, Gaithersburg, MD, USA) following the manufacturer's protocol. Briefly, cells in each group were seeded into 96-well plates at a density of 3,000 cells per well for 24 h. Then, ten microliters of CCK-8 solution were added into each well, and cells were incubated for 1, 2, and 3 d at 37°C. The absorbance at each time point was read at 450 nm with a microplate reader (Bio-Rad, Hercules, CA, USA).

### 2.6. Terminal-Deoxynucleotidyl Transferase-Mediated Nick End Labeling Assay (TUNEL)

The TUNEL assay was used to detect DNA fragmentation in human trophoblast cells according to the instructions of the TUNEL kit. Cell nuclei were stained with DAPI dissolved in PBS for 20 min at room temperature in the dark. Digital images were acquired by a fluorescent microscope (Leica, Germany).

### 2.7. Cell Migration and Invasion Assays

Transwell assay was performed as Chen et al. described [26]. For the cell migration assay, trophoblast cells (3 × 10^4^ cells/well) were seeded on upper Transwell chambers (8-*μ*m pore size, Costar, Cambridge, Massachusetts) with serum-free medium. The lower chambers were filled with medium contained 15% FBS. After 24 h of incubation, the cells that migrated to the lower chambers were fixed with 4% paraformaldehyde and stained with 0.1% crystal violet for 15 min. The number of migratory cells was counted in three randomly selected fields under a light microscope. The procedure of the invasion assay was similar to the migration assay, except that Transwell inserts were precoated with Matrigel (BD Biosciences).

### 2.8. Luciferase Reporter Assay

The potential wild-type (WT) sequence in the 3′-untranslated regions (3′-UTRs) of human FOXO3 and mutant (MUT) sequence in the miR-155 target site were inserted into the pmiR-RB-Report control vector (Promega Corporation) to generate the recombinant vectors, pmiR-WT, and pmiR-MUT, respectively. Next, the constructed recombined vectors (0.8 *μ*g) were cotransfected with miR-155 mimic or NC (100 nM) into 293 T cells (2 × 10^5^) at 37°C. After 48 h of further incubation, the cells were lysed, and the luciferase activity was analyzed with the Dual-Luciferase reporter assay kit (Promega Corporation) according to the manufacturer's instructions.

### 2.9. Establishment of a Rat Model of PE

Adult Sprague-Dawley rats (14-16 weeks old), 50 female and 50 male rats, were purchased from Nanjing Junke Biological Co., LTD. At a ratio of 1 : 2, male and female rats were caged together during oestrus at a temperature of 18-28°C and 40-70% relative humidity. Then, pregnant SD rats were randomly assigned into three groups: the Sham group, the PE group, and the miR-155 inhibitor group (ten rats per group). The PE rat model was established by subcutaneous injection of NG-nitro-L-arginine methyl ester at a dose of 200 mg/kg/day for seven days. In the miR-155 inhibitor group, rats with the PE model were intravenous injected with the miR-155 inhibitor intravenously. Rats in the sham group were subcutaneous administrated with the same volume of normal saline. On day 20 of pregnancy, rats were killed, and samples of blood and placentas were obtained for further analysis. Animal experiments were approved by the Animal Care and Use Committee of The Third Affiliated Hospital of Zhengzhou University in accordance with the Guidelines for use and care of animals.

### 2.10. Enzyme-Linked Immunosorbent Assay (ELISA)

The serum or cell supernatant levels of the inflammatory cytokines, including inflammatory cytokines interleukin-6 (IL-6), interleukin-8 (IL-8), and tumor necrosis factor-alpha (TNF-*α*), as well as MMP2 were analyzed using commercially available high sensitivity ELISA kits (R&D Systems Europe, Ltd.). Their concentrations were measured in accordance with their corresponding standard curves. Experiments were independently performed in triplicate.

### 2.11. Reverse Transcription Quantitative PCR (RT-qPCR)

Total RNA was isolated from tissue samples or cell lines with TRIzol Reagent (Invitrogen, USA). RNA was reverse transcribed into cDNA using the miScript Reverse Transcription II kit or the PrimeScript RT Master Mix kit (QIAGEN, Hilden, Germany). RT-qPCR analysis was carried out with the SYBR Green qPCR assay (Thermo Fisher Scientific, Massachusetts, USA) and gene-specific primers. The relative gene expression levels were calculated using the 2^-∆∆Ct^ method with U6 (for miRNA) or GAPDH (for mRNA) as the internal control.

### 2.12. BSP Assay

BSP was performed to evaluate miR-155 methylation levels. In brief, genomic DNA was isolated from the tissue samples using a PureLink™ Genomic DNA Mini Kit (Thermo Fisher, USA) according to the manufacturer's instructions. Sodium bisulphite modification of the DNA was performed using an EZ DNA Methylation-Gold Kit (Zymo Research, USA). PCRs (primer sequences: forward: GGTTGTGTTTGAGAATAAAGGGG; reverse: CCAACCAATATAACTCGCCCT) were performed with a ZymoTaq™ PreMix (Zymo Research, USA) under the following conditions: 90°C for 10 min; 50 cycles of 95°C for 30 s, 54°C for 35 s, and 72°C for 30 s; and an elongation step of 72°C for 5 min. The PCR products were ligated into T-vectors and subsequently subjected to blue-white screening. After the selected monoclonal clones were sequenced, the methylation levels of the sequences were analyzed.

### 2.13. Western Blot Analysis

Extraction of total protein sample was performed with RIPA lysis buffer (Beyotime Biotechnology, Shanghai, China), and the protein concentration was detected using a BCA assay kit (Beyotime Biotechnology) according to the manufacturer's instruction. Then protein samples (30 *μ*g) were separated by 10% sodium dodecyl sulfate-polyacrylamide gel electrophoresis (SDS-PAGE) and transferred onto PVDF membranes. After blocking with 5% nonfat milk for 2 h at room temperature, the membranes were incubated overnight at 4°C with primary antibodies against CytC, MMP2, SOD1, FOXO3, and GAPDH (Abcam, Cambridge, MA) and then interacted with horseradish peroxidase- (HRP-) conjugated secondary antibodies (Abcam) for 2 h at room temperature. Specific immunoreactive bands were detected and visualized by the Chemiluminesce ECL reagent (Perkin Elmer, Waltham, MA).

### 2.14. Immunohistochemical Assay

The placental tissues were fixed in 4% paraformaldehyde (Sigma, MO, USA) and embedded in paraffin wax. Paraffin-embedded tissues were cut into 5 *μ*m thick slices. Then, the tissue slices were routinely dewaxed, washed with PBS, and soaked in 0.01 M citrate buffer (pH = 6.0). After antigen recovery with a microwave, the slices were washed with PBS and maintained with 3% hydrogen peroxide for 20 min. Next, the slices were incubated with anticytokeratin 7 (CK7) and anti-MMP2 (Abcam, Cambridge, MA, USA) at 4°C overnight. Subsequently, the slices were incubated with goat anti-rabbit immunoglobulin G secondary antibodies (Abcam) at 37°C for 30 min. The staining results were observed under an inverted fluorescent microscope.

### 2.15. Statistical Analysis

The SPSS statistical software package (SPSS Inc., Chicago, IL, USA) was used for all statistical analyses. Data were expressed as mean ± standard deviation of three independent tests. One-way analysis of variance (ANOVA), followed by Turkey's post hoc test, was used to compare the results among multiple groups with values of *P* less than 0.05 as statistically significant.

## 3. Results

### 3.1. Occurrence of PE Was Associated with Elevated Expression of miR-155

**Figure 1:**
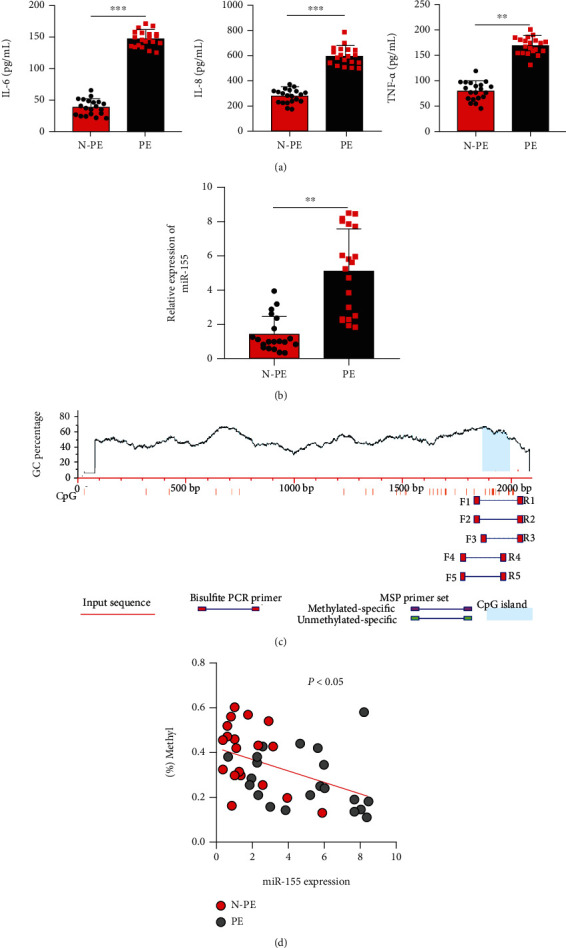
Inflammatory response and upregulated miR-155 expression are observed in PE patients. (a) IL-6, IL-8, and TNF-*α* in the serum derived from 20 cases of PE and 20 cases of non-PE samples were detected using enzyme-linked immuno-sorbent assay. (b) miR-155 expression in the placental tissues of PE patients or normal placental tissues determined by RT-qPCR (*n* = 20). (c) A schematic drawing shows CpG sites in the miR-155 gene promoter and BSP assay location. Each vertical bar represents a CpG site (12 sites). (d) miR-155 expression was inversely correlated with its promoter methylation level in 20 cases of PE and 20 age-matched non-PE samples. ∗*P* < 0.05, and ∗∗*P* < 0.01.

We initially investigated the inflammatory reaction in serum samples derived from PE patients. The ELISA data revealed that the levels of IL-6, IL-8, and TNF-*α* in the PE group were significantly higher compared with those in the control group (Figure 1(a)). To better understand the role of miR-155 in PE, RT-qPCR was used to analyzed its expression level, which showed that the expression of miR-155 was remarkably elevated in the placental tissues of PE patients compared to normal placental tissues (Figure 1(b)). By using BSP, promoter DNA methylation of miR-155 was studied in placental tissues. The representative results of methylation levels of CpG sites in miR-155 genomic loci are shown in Figure 1(c). Moreover, the levels of DNA methylation were inversely correlated with the miR-155 expression level in placental tissues (Figure 1(d)). These data demonstrated that dysregulated miR-155 expression associated with promoter DNA methylation may play an important role in the pathogenesis of PE.

### 3.2. miR-155 Regulated Cell Viability, Apoptosis, Mobility, and Oxidative Stress in Trophoblast Cells

**Figure 2:**
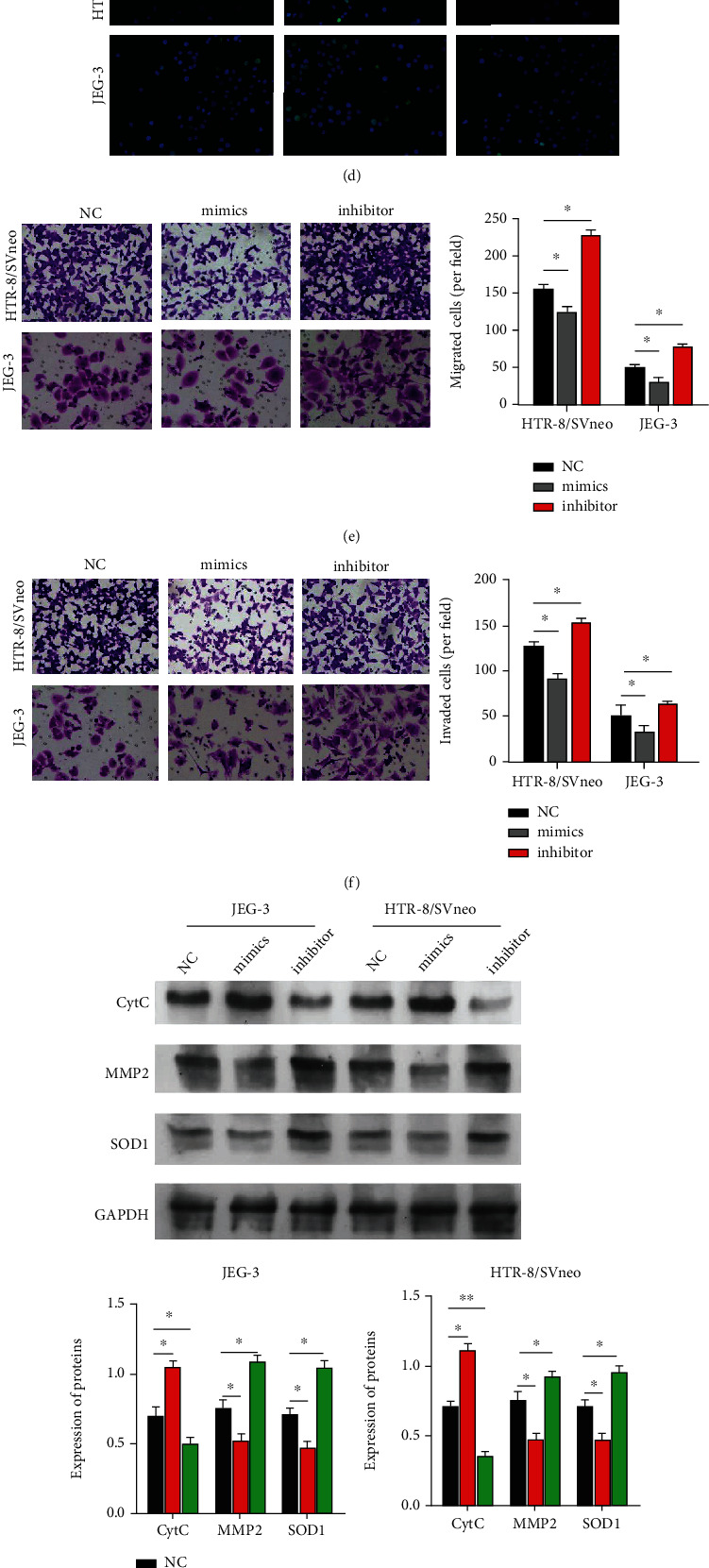
miR-155 regulated cell viability, apoptosis, mobility, and oxidative stress in trophoblast cells. Negative control (NC), miR-155 mimic, and the miR-155 inhibitor were transfected into HTR8/SVneo and JEG-3 cells. (a) RT-qPCR assay was used to measure the expression of miR-155 in the cells. (b, c) Cell Counting Kit-8 (CCK-8) assay was used to measure the cell viability after transfection in HTR8/SVneo and JEG-3 cells. (d) Detection of cell apoptosis by TUNEL assay. The nuclei of apoptotic cells had green fluorescence. Hoechst staining showed that the nuclei of placental trophoblast cells were blue-violet. Microscopic images and quantitative analysis of Transwell migration (e) and invasion (f) in cells following transfection. (g) Western blot analysis was performed to determine the expression levels of proteins associated with apoptosis (CytC), invasion (MMP2), and oxidative stress (SOD1). (h) RT-qPCR assay and (i) ELISA were used to measure the expression of MMP2 in the cells. ∗*P* < 0.05, ∗∗*P* < 0.01, and ∗∗∗*P* < 0.001.

To investigate the functional role of miR-155 in trophoblast cells, two trophoblast cell lines (HTR-8/SVneo and JEG-3) were transfected with miR-155 mimic or the inhibitor for gain-of-function or loss-of-function assays, respectively. Transfection results demonstrated that miR-155 expression was significantly increased after miR-155 mimic transfection, while it was remarkedly decreased after miR-155 inhibitor transfection in HTR-8/SVneo and JEG-3 cells (Figure 2(a)). The results from the CCK-8 assay showed that overexpressed miR-155 inhibited cell viability, whereas silenced miR-155 promoted cell viability in HTR-8/SVneo (Figure 2(b)) and JEG-3 (Figure 2(c)) cells. Consistently, TUNEL staining indicated that miR-155 mimic could significantly elevate the amounts of apoptotic cells, while transfection with the miR-155 inhibitor reduced the amounts of apoptotic cells (Figure 2(d)). According to the Transwell assay, overexpressed miR-155 led to a measurable decrease in migration (Figure 2(e)) and invasion (Figure 2(f)) of the HTR8/SVneo and JEG-3 cells, whereas the effects of poorly expressed miR-155 on HTR8/SVneo and JEG-3 cells had an opposite trend. Furthermore, Western blot analysis (Figure 2(g)) revealed that upregulated miR-155 increased the expression level of apoptotic-related CytC, while it suppressed the expression levels of antioxidant-related SOD1 and invasion-related MMP2. It was noted that the effects of the miR-155 inhibitor on HTR8/SVneo and JEG-3 cells had an opposite trend compared with the effects of overexpressed miR-155. In addition, RT-qPCR (Figure 2(h)) and ELISA assay (Figure 2(i)) further validated the expression levels of MMP2 under miR-155 mimic or inhibitor transfection.

### 3.3. 5-Aza Treatment Attenuated the Effects of miR-155 Knockdown on Trophoblast Dysfunction In Vitro

**Figure 3:**
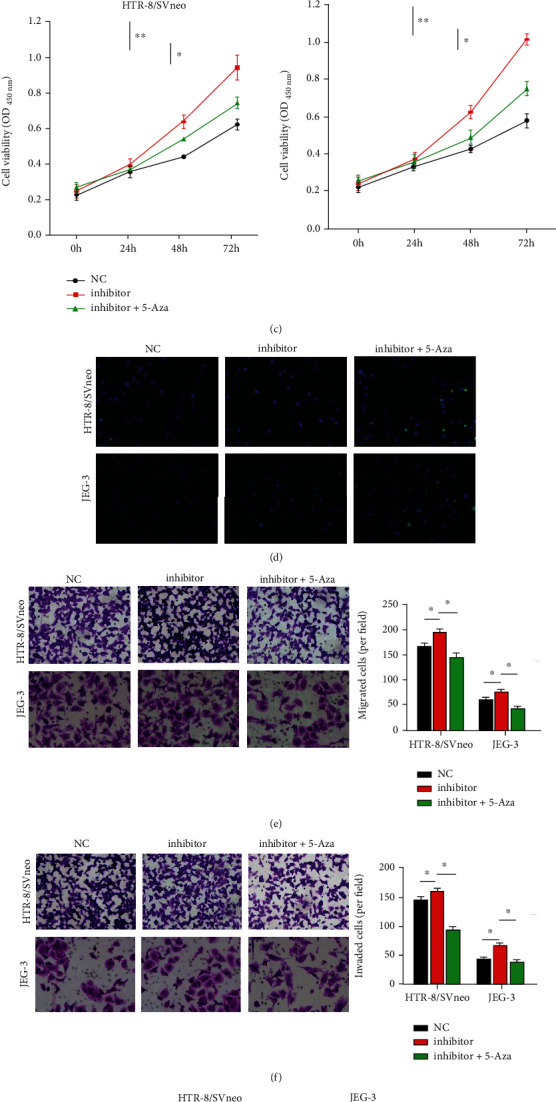
5-Aza treatment attenuated the effects of miR-155 knockdown on trophoblast dysfunction *in vitro*. (a) RT-qPCR analysis of miR-155 indicated that the miR-155 inhibitor-evoked downregulation of miR-155 was reversed by 5-Aza treatment of HTR8/SVneo and JEG-3 cells. (b) 5-Aza inhibited methylation level of miR-155 promoter in HTR8/SVneo and JEG-3 cells. (c) CCK-8 assay indicated that 5-Aza reversed the promotion of cell viability by the miR-155 inhibitor in HTR8/SVneo and JEG-3 cells. (d) Detection of cell apoptosis by TUNEL assay. Transwell assays indicated that 5-Aza attenuated the increased cell migration (e) and invasion (f) induced by the miR-155 inhibitor (magnification 200×) in HTR8/SVneo and JEG-3 cells. (g) The protein levels of MMP2, CytC, and SOD1 were measured in 5-Aza-treated HTR8/SVneo and JEG-3 cells with the miR-155 inhibitor. ∗*P* < 0.05, ∗∗*P* < 0.01, and ∗∗∗*P* < 0.001.

Based on the result that miR-155 was silenced by DNA methylation in placental tissues, we speculated that miR-155-mediated regulation might be involved in DNA methyltransferase inhibitor, 5-Aza-induced miR-155 upregulation in trophoblast cells. We transfected 5-Aza-treated HTR8/SVneo and JEG-3 cells with the miR-155 inhibitor. At first, it was found that the miR-155 inhibitor-induced downregulation of miR-155 expression was strongly reversed by 5-Aza treatment in both HTR8/SVneo and JEG-3 cells (Figure 3(a)). Results of BSP showed that there was no significant difference in the methylation level of miR-155 promoter region between the control group and inhibitor treatment group, but the methylation level decreased significantly after 5-Aza treatment (Figure 3(b)). Subsequently, a series of functional experiments demonstrated that miR-155 inhibitor transfection elevated cell viability (Figure 3(c)) and inhibited cell apoptosis (Figure 3(d)), whereas it enhanced cell migration (Figure 3(e)) and invasion (Figure 3(f)) of HTR8/SVneo and JEG-3 cells, which were all significantly reversed by cotreatment with 5-Aza. Moreover, 5-Aza treatment abolished the regulation of the miR-155 inhibitor on the protein expression levels of MMP2, CytC, and SOD1 in HTR8/SVneo and JEG-3 cells (Figure 3(g)).

### 3.4. miR-155 Binds and Negatively Regulates FOXO3 Expression

**Figure 4:**
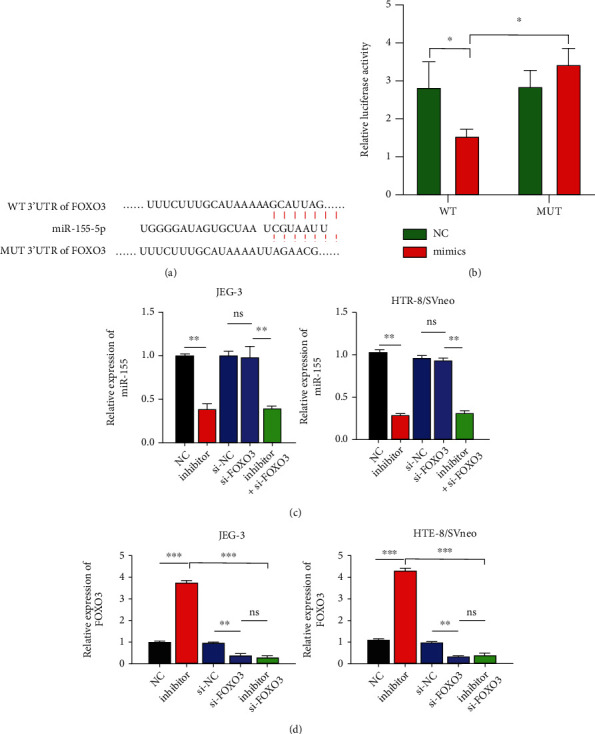
miR-155 binds and negatively regulates FOXO3 expression. (a) The binding site between miR-155 and FOXO3. (b) Relative luciferase activity was measured in 293 T cells cotransfected with miR-155 mimics and WT FOXO3 or MUT FOXO3. HTR8/SVneo and JEG-3 cells were transfected with NC, inhibitor, si-NC, si-FOXO3, and inhibitor + si-FOXO3, respectively. RT-qPCR assay was used to determine the expression of miR-155 (c) and FOXO3 (d) mRNA in the above-transfected cells. ∗*P* < 0.05, and ∗∗*P* < 0.01. ns: not significant.

Identification of target genes is an essential step to study the association between miRNA and disease. Previous studies indicated that miR-155 regulates FOXO3 expression in various cancers. To verify whether miR-155 and FOXO3 have a similar binding in trophoblast cells, we first confirmed that there were complementary binding sites between miR-155 and FOXO3 using TargetScan Human version 7.2 (Figure 4(a)). Then, the luciferase reporter assay was used to further investigate the interaction. The results showed that the luciferase activity in 293 T cells transfected with miR-155 mimic was significantly lower than that in cells transfected with the NC (Figure 4(b)). Additionally, the effect of miR-155 on FOXO3 expression was also determined using RT-qPCR analysis. We first confirmed that miR-155 expression was downregulated after miR-155 inhibitor transfection in HTR8/SVneo and JEG-3 cells (Figure 4(c)). Then, it was observed that si-FOXO3 transfection caused a decrease in the FOXO3 mRNA level. Moreover, si-FOXO3 transfection reversed the suppression of FOXO3 expression in miR-155 inhibitor-transfected HTR8/SVneo and JEG-3 cells (Figure 4(d)). The present results indicated that miR-155 directly binds and inhibits FOXO3 expression in trophoblast cells.

### 3.5. miR-155 Knockdown Ameliorated Trophoblast Dysfunction In Vitro by Upregulating FOXO3

**Figure 5:**
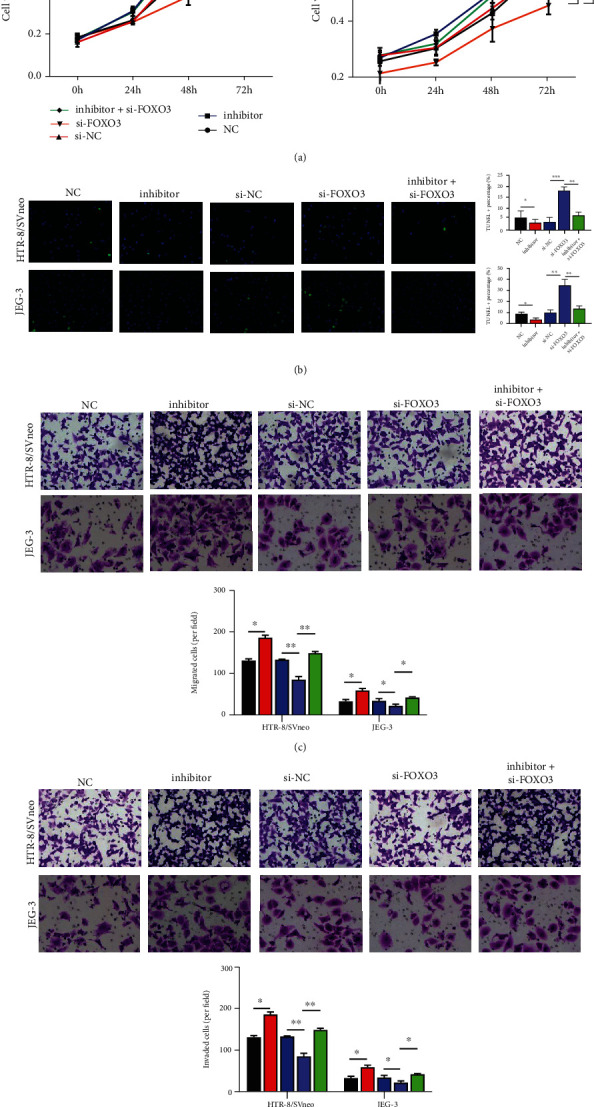
miR-155 knockdown increased cell viability, migration, and invasion in trophoblast cells by upregulating FOXO3. HTR8/SVneo and JEG-3 cells were transfected with NC, inhibitor, si-NC, si-FOXO3, and inhibitor + si-FOXO3, respectively. (a) Cell viability was evaluated by CCK-8 assay in the above-transfected cells. (b) Detection of cell apoptosis by TUNEL assay. Transwell assay was used to analyze cell migration (c) and invasion (d) in the above-transfected cells. ∗*P* < 0.05, and ∗∗*P* < 0.01.

**Figure 6:**
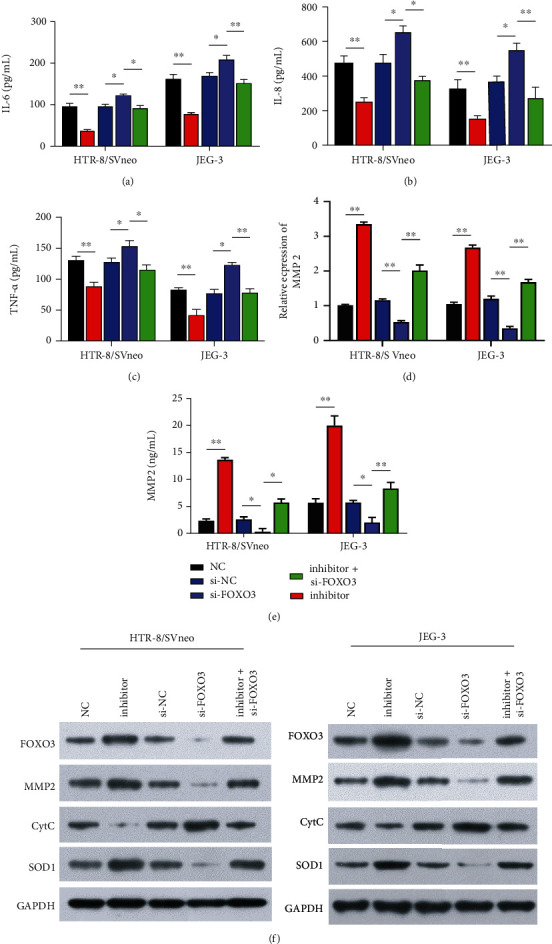
miR-155 knockdown inhibited inflammation, apoptosis, and oxidative stress in trophoblast cells by upregulating FOXO3. HTR8/SVneo and JEG-3 cells were transfected with NC, inhibitor, si-NC, si-FOXO3, and inhibitor + si-FOXO3, respectively. ELISA assay was used to measure the release of (a) IL-6, (b) IL-8, and (c) TNF-*α* in cell supernatants of the above-transfected cells. (d, e) Expression levels of MMP2 were determined using RT-qPCR assay (d) and ELISA assay (e) in the above-transfected cells. (f) The protein levels of FOXO3, MMP2, CytC, and SOD1 were measured in the above-transfected cells. ∗*P* < 0.05, and ∗∗*P* < 0.01.

To further investigate the role of miR-155/FOXO3 signaling in PE, we transfected trophoblast cells with si-FOXO3 together with or without the miR-155 inhibitor and evaluated their effects on cell functions. The results of the CCK-8 assay showed that knockdown of FOXO3 decreased cell viability of HTR8/SVneo and JEG-3 cells, while the miR-155 inhibitor rescued the effect of si-FOXO3 (Figure 5(a)). TUNEL staining further confirmed the promotion of apoptosis by FOXO3 knockdown, which was reversed by the miR-155 inhibitor (Figure 5(b)). Additionally, we observed lower migration (Figure 5(c)) and invasion (Figure 5(d)) abilities of HTR8/SVneo and JEG-3 cells transfected with si-FOXO3 compared with cells transfected with si-NC, whereas the miR-155 inhibitor reversed the effects of FOXO3 knockdown. By collecting the cell supernatants of trophoblasts from different groups, we analyzed the effects of miR-155/FOXO3 signaling on the levels of the inflammatory cytokines. The results from ELISA assay showed that FOXO3 knockdown alone increased the release of IL-6 (Figure 6(a)), IL-8 (Figure 6(b)), and TNF-*α* (Figure 6(c)), while the miR-155 inhibitor rescued the suppressive effects of FOXO3 knockdown on these inflammatory cytokines. In addition, RT-qPCR (Figure 6(d)) and ELISA assay (Figure 6(e)) consistently demonstrated that the miR-155 inhibitor reversed the decreased expression levels of MMP2 induced by FOXO3 knockdown in HTR8/SVneo and JEG-3 cells. Furthermore, Western blot analysis further supported that the regulatory roles of FOXO3 knockdown on the protein expression levels of FOXO3, MMP2, CytC, and SOD1 were notably reversed by the miR-155 inhibitor (Figure 6(f)).

### 3.6. Therapeutic Effects of the miR-155 Inhibitor in the PE Rat Model

**Figure 7:**
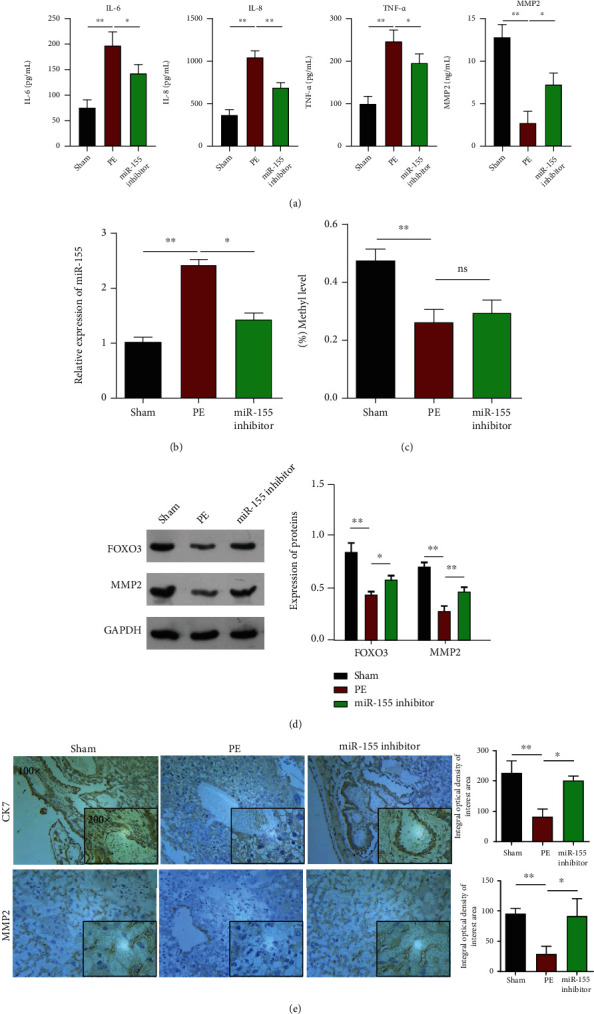
Therapeutic effects of the miR-155 inhibitor in the PE rat model. Pregnant SD rats were randomly dived into three groups: the sham group (*n* = 10), the PE group (*n* = 10), and the PE + miR − 155 inhibitor group (*n* = 10). (a) ELISA assay was utilized to measure the release of IL-6, IL-8, TNF-*α*, and MMP2 in the samples of blood from PE rat model. (b) RT-qPCR assay was used to determine expression of miR-155 in the samples of blood from the PE rat model. (c) BSP was performed to evaluate miR-155 methylation levels in the samples of blood from the PE rat model. (d) The protein levels of FOXO3 and MMP2 were measured in the placental tissues from the PE rat model. (e) Immunohistochemistry assay was performed in paraffin sections of human placentas to show the expression levels of CDK7 and MMP2. ∗*P* < 0.05, and ∗∗*P* < 0.01.

ELISA assay (Figure 7(a)) showed a higher concentration of IL-6, IL-8, TNF-*α*, and MMP2 in the placentas of PE rats than in the placentas of sham rats. The injection of the miR-155 inhibitor significantly prevented an increase in the concentration of IL-6, IL-8, TNF-*α*, and MMP2 in the placentas of PE rats. Consistent with the *in vitro* data, the miR-155 inhibitor caused a reduction in miR-155 expression in PE rats (Figure 7(b)), which could be explained by higher DNA methylation levels in PE rats, compared with the miR-155 inhibitor group (Figure 7(c)). Western blot analysis indicated that the injection of the miR-155 inhibitor abated the reduction in FOXO3 and MMP2 protein levels in PE rats (Figure 7(d)). The immunohistochemical assay results also revealed that there were considerable amounts of brown particles in the miR-155 inhibitor group, which represented a positive expression of CK7 and MMP2 (Figure 7(e)).

## 4. Discussion

The exact pathogenesis of PE is not yet fully understood, and intense research efforts are focused on PE to elucidate the pathophysiological mechanisms, of which miRNA regulatory networks play an important role in diverse pathological processes [27]. Here, it was demonstrated that miR-155 is significantly upregulated in the placental tissues from PE patients compared with the control group. With the upregulation of miR-155, the serum levels of IL-6, IL-8, and TNF-*α* in PE group were significantly higher compared with those in the control group, which could be explained by the fact that PE is a systemic inflammatory disorder [28]. Consistently, miR-155 expression exhibited a significant increase with antiangiogenic, inflammatory, and apoptotic functions in maternal plasma at the time of severe PE compared to time-matched controls [29]. NF-*κ*B-induced miR-155 induces vascular smooth muscle cell dysfunction, leading to PE hypertension [30]. Next, we used gain-of-function and loss-of-function assays to further validate the role of miR-155 in trophoblasts. The data showed that overexpression of miR-155 significantly suppressed cell viability, migration, and invasion, while promoting apoptosis, inflammation, and oxidative stress. Knockdown of miR-155 obtained the opposite results. In line with our data, inhibiting miR-155 increased the production of Hedgehog ligand sonic hedgehog (SHH) and improved the phenotype in primary trophoblasts from patients with PE [31]. miR-155 inhibits cell invasion and expression of eNOS in trophoblast cells [17]. In addition, miR-155 inhibits the proliferation and invasion of HTR-8/SVneo cells by downregulating cyclin D1 [18]. While in this study, we further confirmed that miR-155 upregulated CytC expression, while it downregulated the expression levels of MMP2 and SOD1. These data supported that miR-155 promotes apoptosis and oxidative stress in trophoblast cells.

Epigenetic mechanisms such as DNA methylation and histone modifications play major roles in the regulation of miRNA expression [32]. Aberrations of both DNA methylation and miRNA expression are very specific depending on cell differentiation and tissue types. Using BSP, our results revealed that expression of miR-155 is inversely correlated with DNA methylation levels, indicating that decreased miR-155 expression is ascribed to a higher DNA methylation level of the promoter region in PE tissues. Unlike mutations, DNA methylation can be reversed, which is similar to other physiological biochemical modifications [33]. Next, we used an approved demethylating agent 5-Aza to treat trophoblast cells, followed by miR-155 inhibitor transfection. As expected, 5-Aza treatment attenuated the effects of miR-155 knockdown on trophoblast cell functions and protein levels of MMP2, CytC, and SOD1 *in vitro*. Based on these facts, we thus speculate that methylation mediated silencing of miR-155 could be an important mechanism contributing to apoptosis, inflammation, and oxidative stress in PE.

Moreover, our results also demonstrated that miR-155 targets the 3′-UTR of FOXO3 mRNA and downregulates its expression in trophoblast cells. In fact, the FOXO family has been reported to be involved in the pathogenesis of PE. For example, abnormal FOXO1 expression may contribute in part to the abnormal trophoblast differentiation in mild PE [34]. Elevated expression of hypoxia-inducible factor-1*α* (HIF-1*α*) increases trophoblastic apoptosis by regulating FOXO3a [35]. Furthermore, the correlation of miR-155 and FOXO3 was previously reported in tumors as follows: expression of miR-155 is upregulated in tumors and acts as an oncogene in cancer [36, 37], while expression of FOXO3 is downregulated and can inhibit the development of tumors [38, 39]. A recent study by Cai et al. [40] not only showed that FOXO3 overexpression promotes trophoblast cell proliferation, migration, invasion, and inhibits apoptosis but also demonstrated that the miR-155/FOXO3 axis is involved in the suppressive effects of Baicalin on the progression of PE. On the basis of these evidences, we further confirmed the therapeutic effects of the miR-155 inhibitor in trophoblast dysfunction, including apoptosis, inflammation, and oxidative stress *in vitro* and PE rat model *in vivo* by upregulating FOXO3.

## 5. Conclusion

Our studies revealed that the DNA methylation-mediated attenuation of miR-155 resulted in FOXO3 overexpression, eventually alleviating increased apoptosis, inflammation, and oxidative stress, as well as increasing cell viability, migration, and invasion in PE *in vitro* and *in vivo*. These findings may provide promising new targets for PE therapy in the future.

## Data Availability

All data generated or analyzed in this study are available in the published article.
